# Supplementary material for: Intraspecific diploidization of a halophyte root fungus drives heterosis
Source: Nat Commun. 2024 Jul 12;15:5872. doi: 10.1038/s41467-024-49468-7 (PMC11245560; doi:10.1038/s41467-024-49468-7)
Supplement: Supplementary file 1 — Supplementary Information [file 41467_2024_49468_MOESM1_ESM.pdf]

Supplementary Materials for

Intraspecific diploidization of a halophyte root fungus drives heterosis

Zhongfeng Li[1,2#], Zhiyong Zhu[1,2,3#], Kun Qian[4,5#], Boping Tang[6#], Baocai Han[7], Zhenhui Zhong[8], Tao Fu[9], Peng Zhou[10*], Eva H. Stukenbrock[11,12], Francis M. Martin[2,13*], Zhilin Yuan[1,2*]

[#] These authors contributed equally

[*] Corresponding authors, E-mail addresses: yuanzl@caf.ac.cn, pzhou@caas.cn and francis.martin@inrae.fr

This PDF file includes:

Figs. S1 to S10
Tables S1 to S6

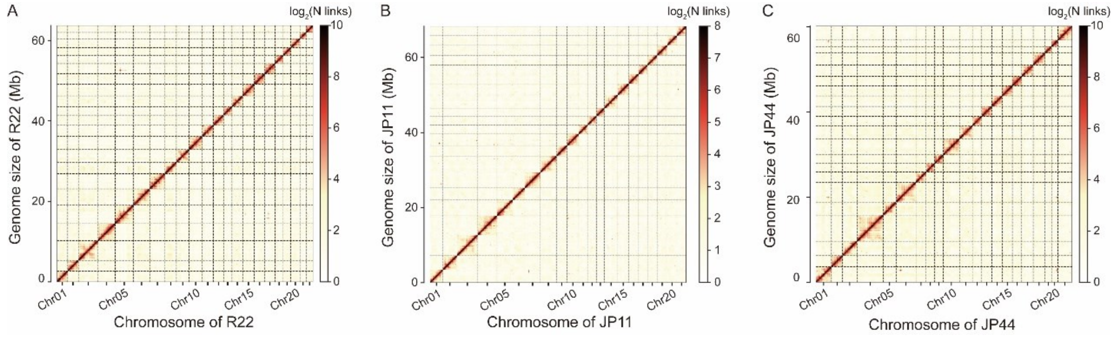

Fig. S1 High correlation of chromosome-scale assembled R22, JP11, and JP44 genomes with Hi-C data. The heatmap represents the normalized contact matrix (20 kb resolution). The strongest and weakest contacts are indicated in black and white, respectively. We used the Hi-C proximity ligation data to anchor the scaffolds onto 22, 21, and 21 chromosomes for R22 (A), JP11 (B), and JP44 (C) genomes, respectively.

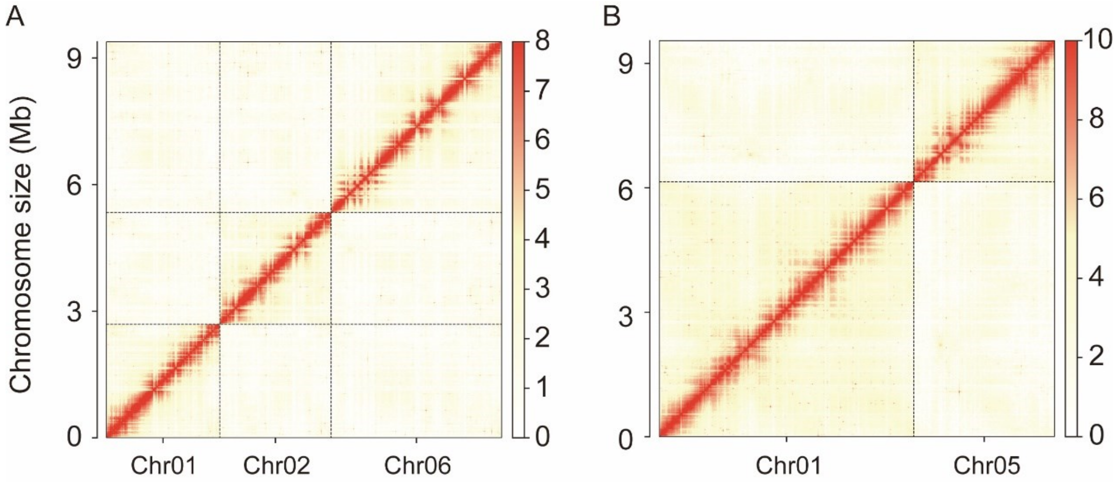

Fig. S2 Enlarged view of Hi-C interaction matrices for chromosomes 1, 2, and 6 in R22 (A) and chromosomes 1 and 5 in JP44 (B). The heatmaps demonstrate that these chromosome groups can be clearly distinguished (20 kb resolution).

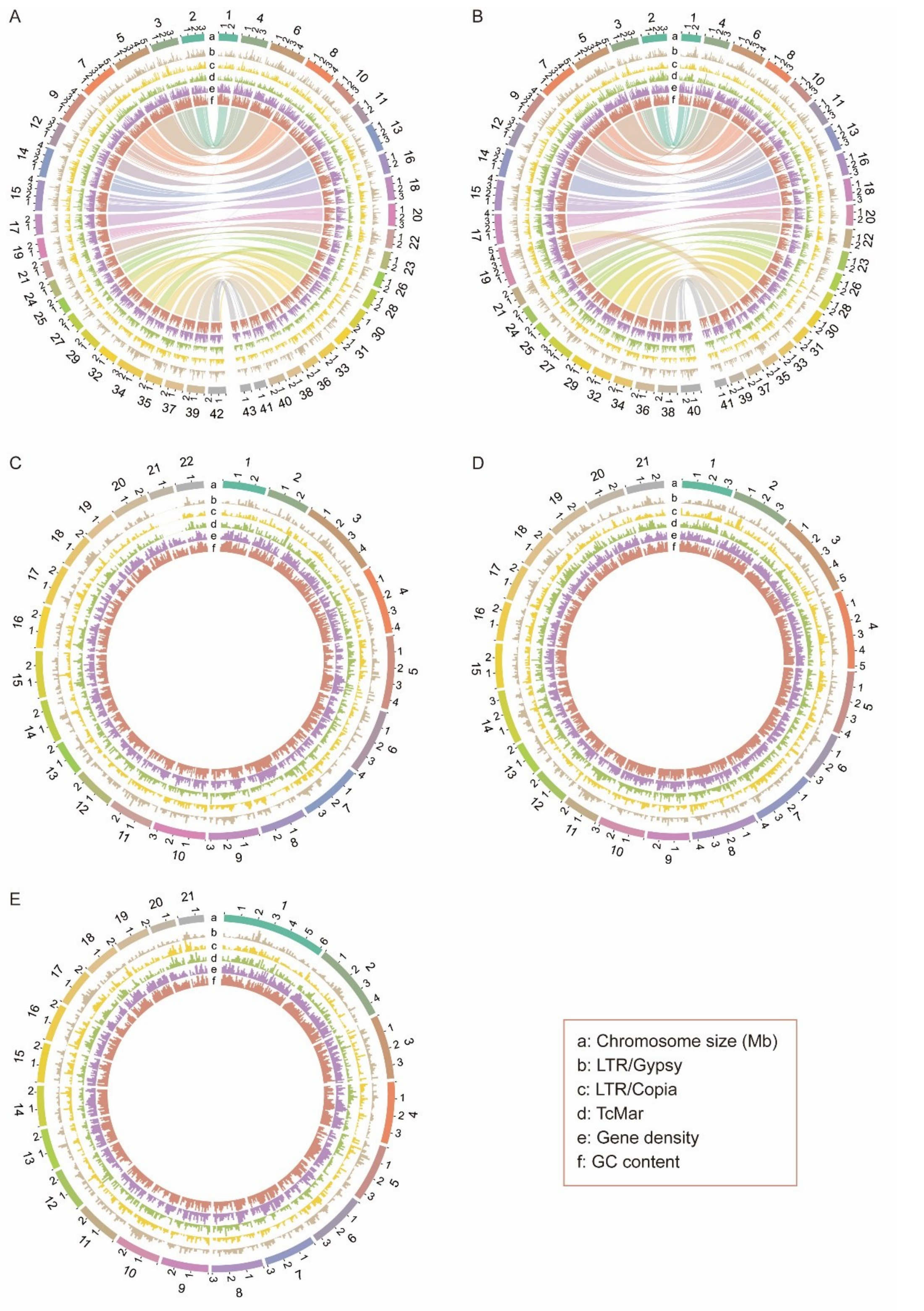

Fig. S3 Circos plots illustrating the genomic landscape of five *L. rhizohalophila* genomes, especially the genome-wide distribution of transposable elements. From outer to inner circles: a, chromosome size; b, LTR/Gypsy; c, LTR/Copia; d, TcMar; e, gene density; and f, GC content. All the densities were calculated using a window size of 100 kb. **(A)** JP19. **(B)** JP8. **(C)** R22. **(D)** JP11. **(E)** JP44. Links in the circle of JP19 and JP8 connect the identified collinear regions between the subgenomes.

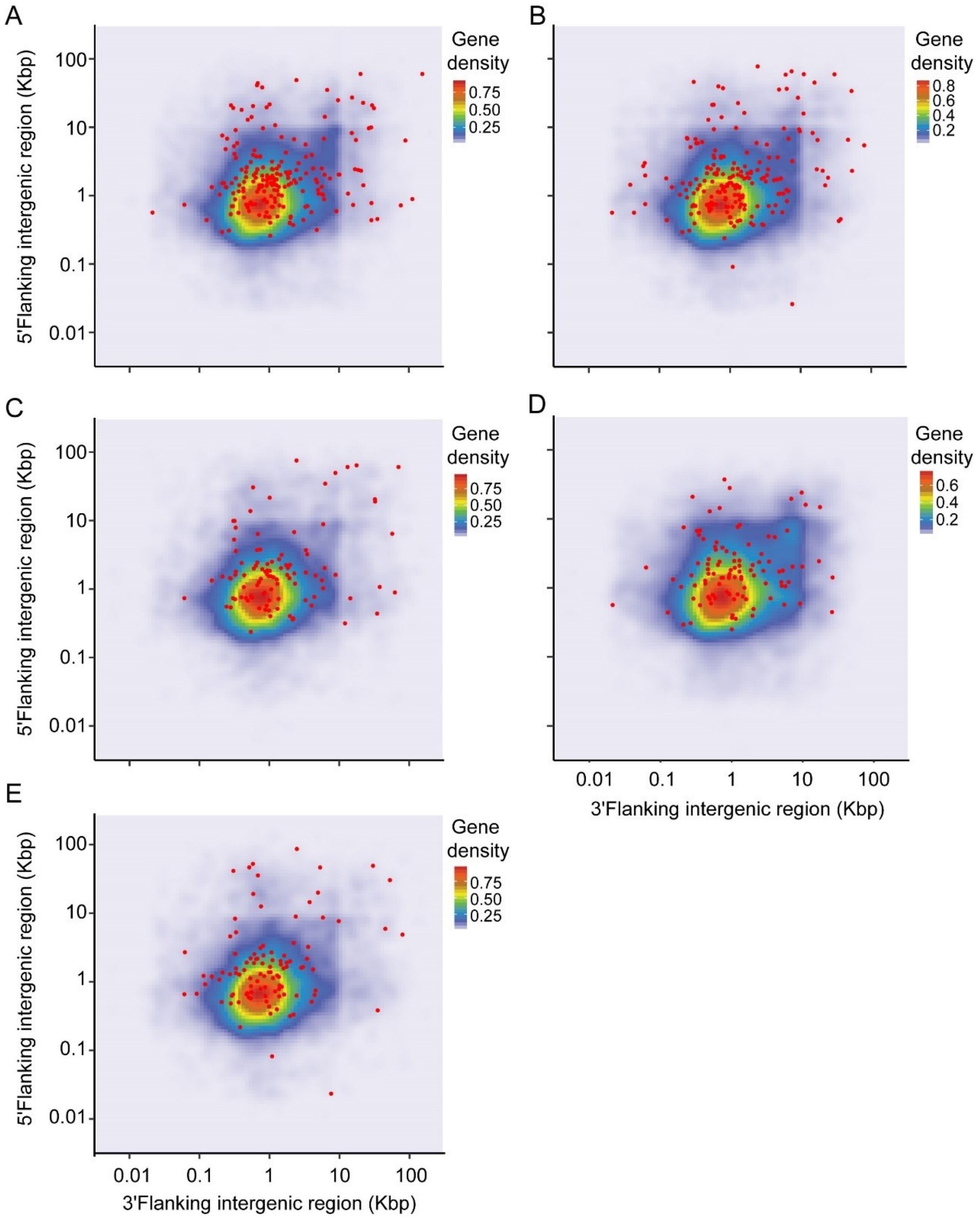

Fig. S4 Density plot illustrating the 5′ and 3′ intergenic distances for the *L. rhizohalophila* genome of JP19 (A), JP8 (B), R22 (C), JP11 (D), and JP44 (E). Distances for all genes are color-coded from blue to red, and the red dots depict the intergenic length of all candidate effector genes, which were identified in silico using EffectorP-fungi 3.0 (https://effectorp.csiro.au/). The results revealed that effector genes are not associated with gene-sparse and repeat-rich genomic compartments, indicating the one-speed genome evolution.

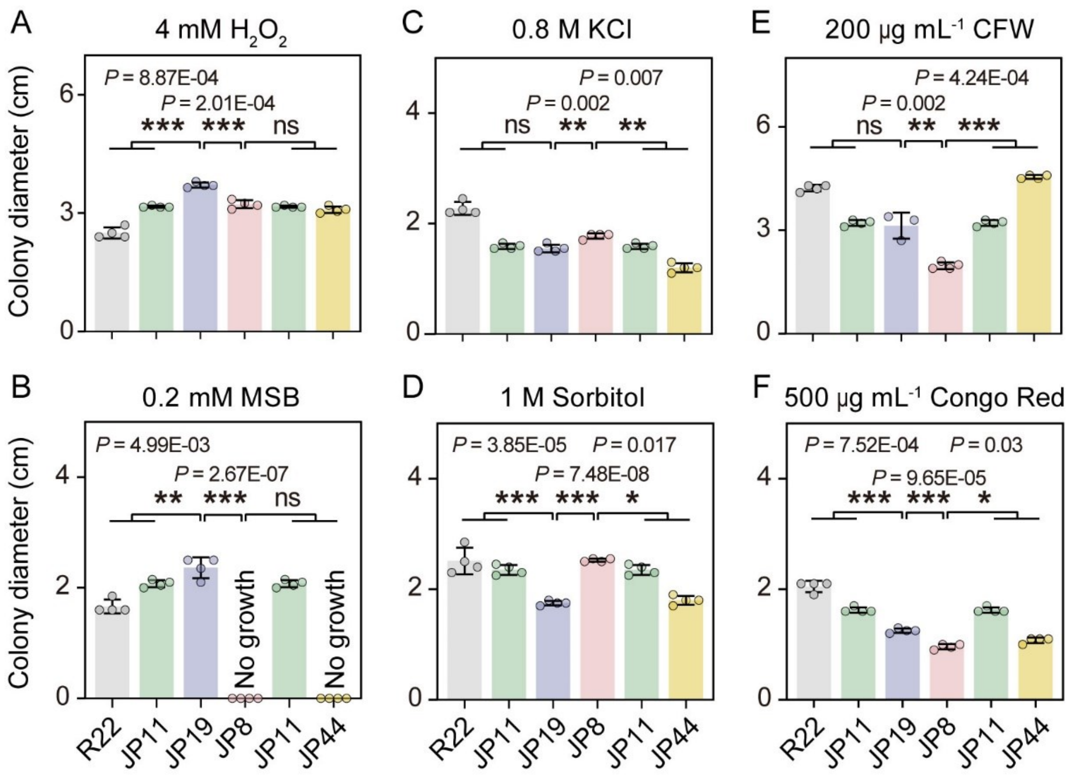

Fig. S5 Growth behavior of five isolates when exposed to a wide range of high abiotic stress conditions. (A-B) Oxidative stress (H_2O_2 and MSB), **(C-D)** osmotic stress (KCl and Sorbitol), and **(E-F)** cell wall stress (CFW and Congo red). Values are means ± SD (n = 4 biologically independent samples). Data were analyzed using one-way analysis of variance (ANOVA). Asterisks indicate significant differences between diploid isolates and their corresponding haploid parents determined by Duncan's test at $P < 0.05$ (***$P < 0.001$, **$P < 0.01$, *$P < 0.05$, ns, $P \geq 0.05$). All the data were repeated three times with similar results. MSB, 2-methyl-1,4-naphthoquinone; CFW, calcofluor white. Source data are provided as a Source Data file.

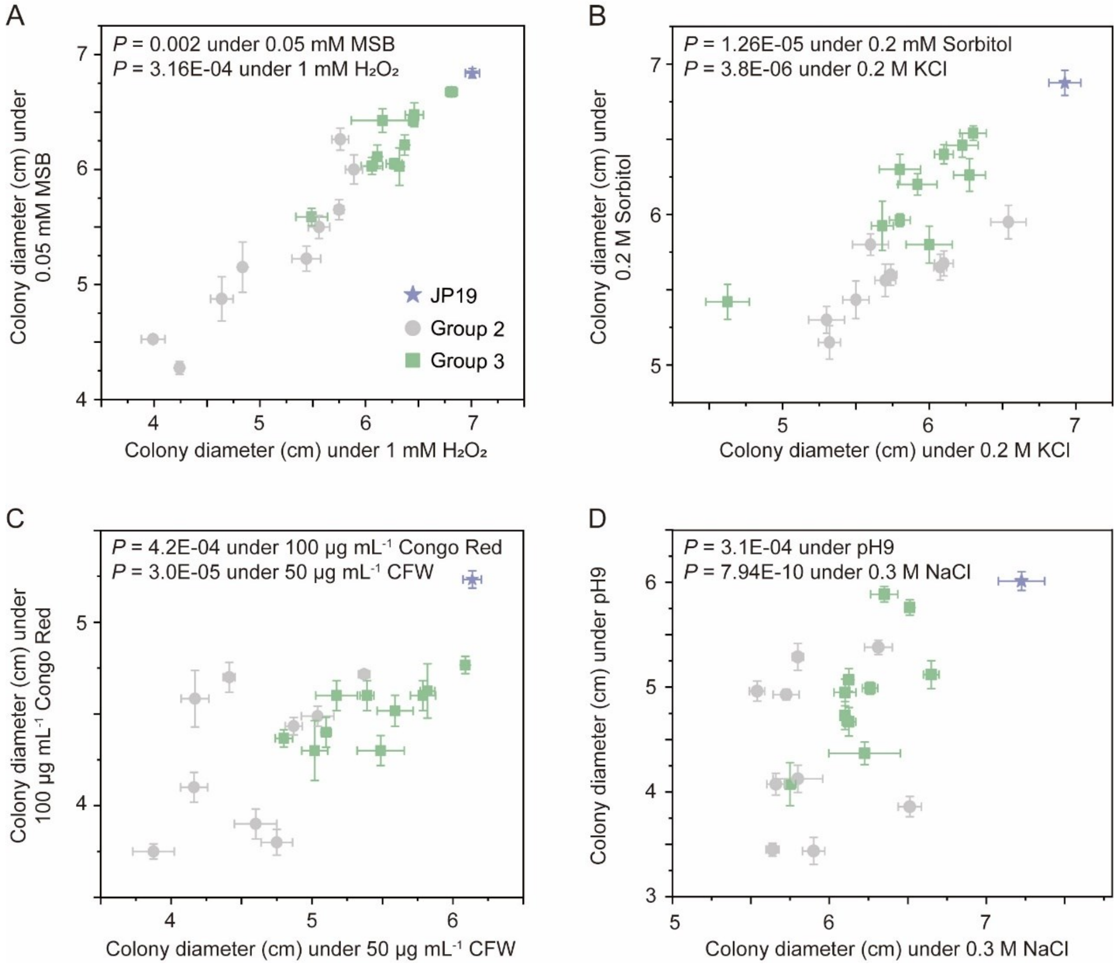

Fig. S6 Comparison of growth behavior between JP19 and all members of its haploid parent groups when exposed to a wide range of abiotic stresses. (A) Oxidative stress (H_2O_2 and MSB). **(B)** Osmotic stress (KCl and sorbitol). **(C)** Cell wall stress (CFW and Congo red staining). **(D)** Saline-alkali stress (0.3 M NaCl and pH9). Values are means ± SD (n = 5 biologically independent samples). Data were analyzed using one-way analysis of variance (ANOVA). *P* value indicate significant differences between JP19 and the two haploid parent groups determined by Duncan's test. Each dot indicates an individual isolate. MSB, 2-methyl-1,4-naphthoquinone; CFW, calcofluor white. All data are representative of at least 5 experimental replicates. Source data are provided as a Source Data file.

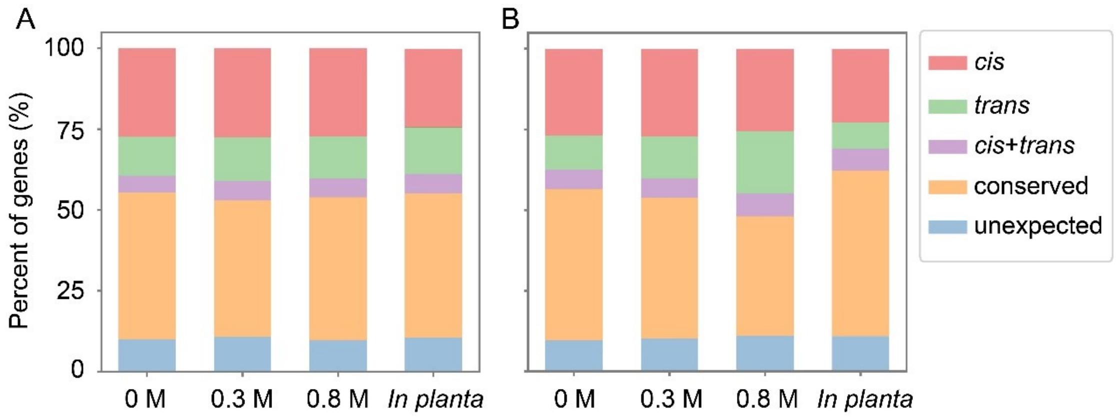

Fig. S7 Regulatory pattern of gene expression in two diploids (A, JP19; B, JP8) under the four growth conditions. Source data are provided as a Source Data file.

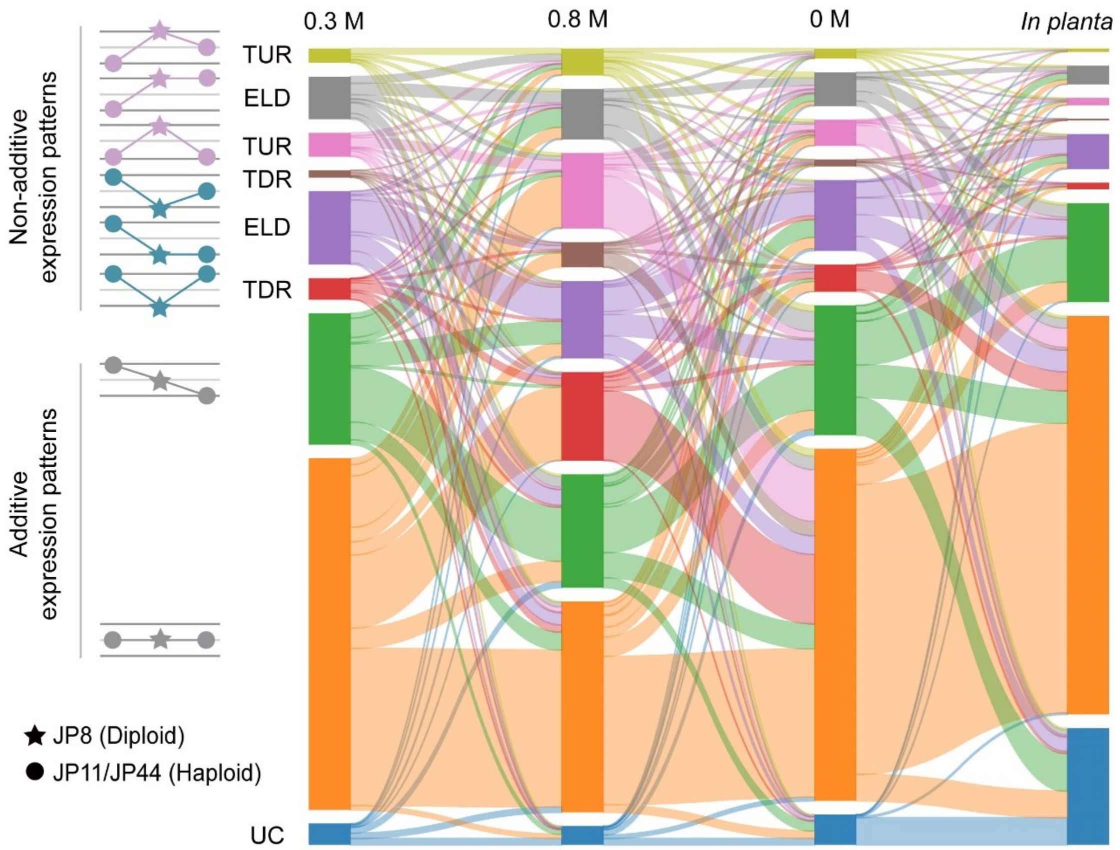

Fig. S8 Alluvial plot illustrating the different categories of additive and non-additive gene expression patterns in JP8 and changes in the number of genes across the four growth conditions. ELD, expression level dominance; TDR, transgressive downregulation; TUR, transgressive upregulation. UC, unclassed. Source data are provided as a Source Data file.

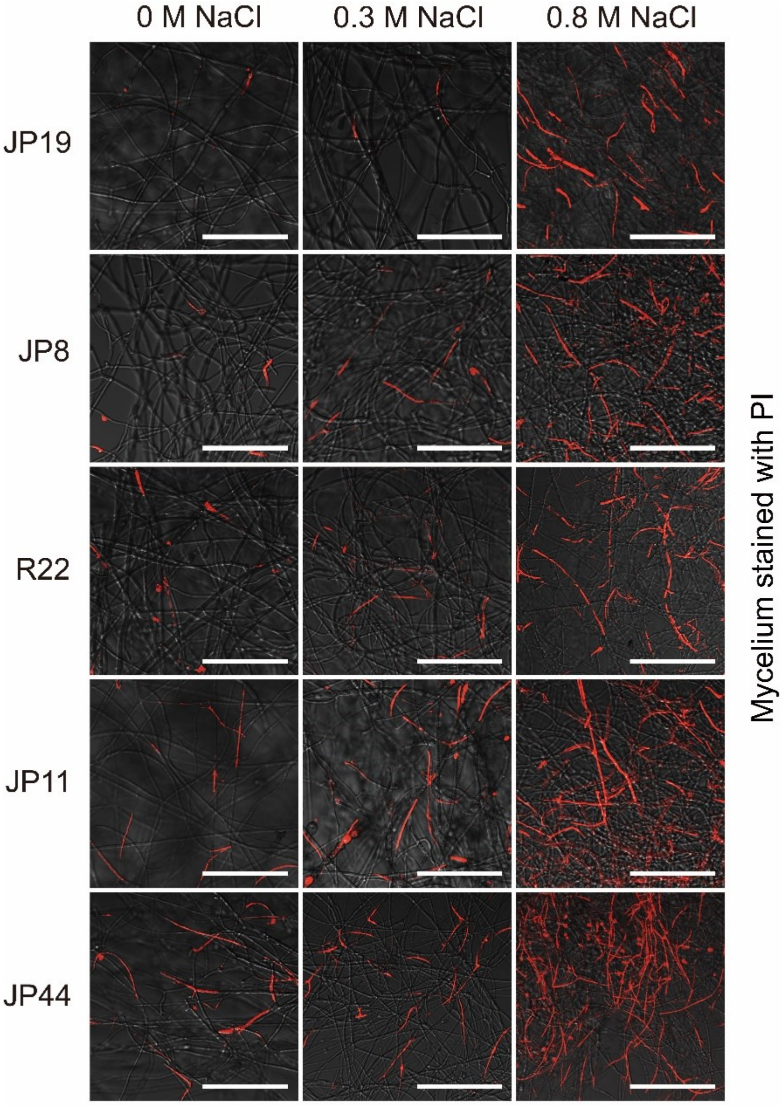

Fig. S9 Mycelial staining using PI under three *in vitro* growth conditions. Increased PI influx into hyphal cells indicates a disturbance in membrane integrity. Scale bars = 50 µm. Images shown are representatives from experiments performed in duplicate.

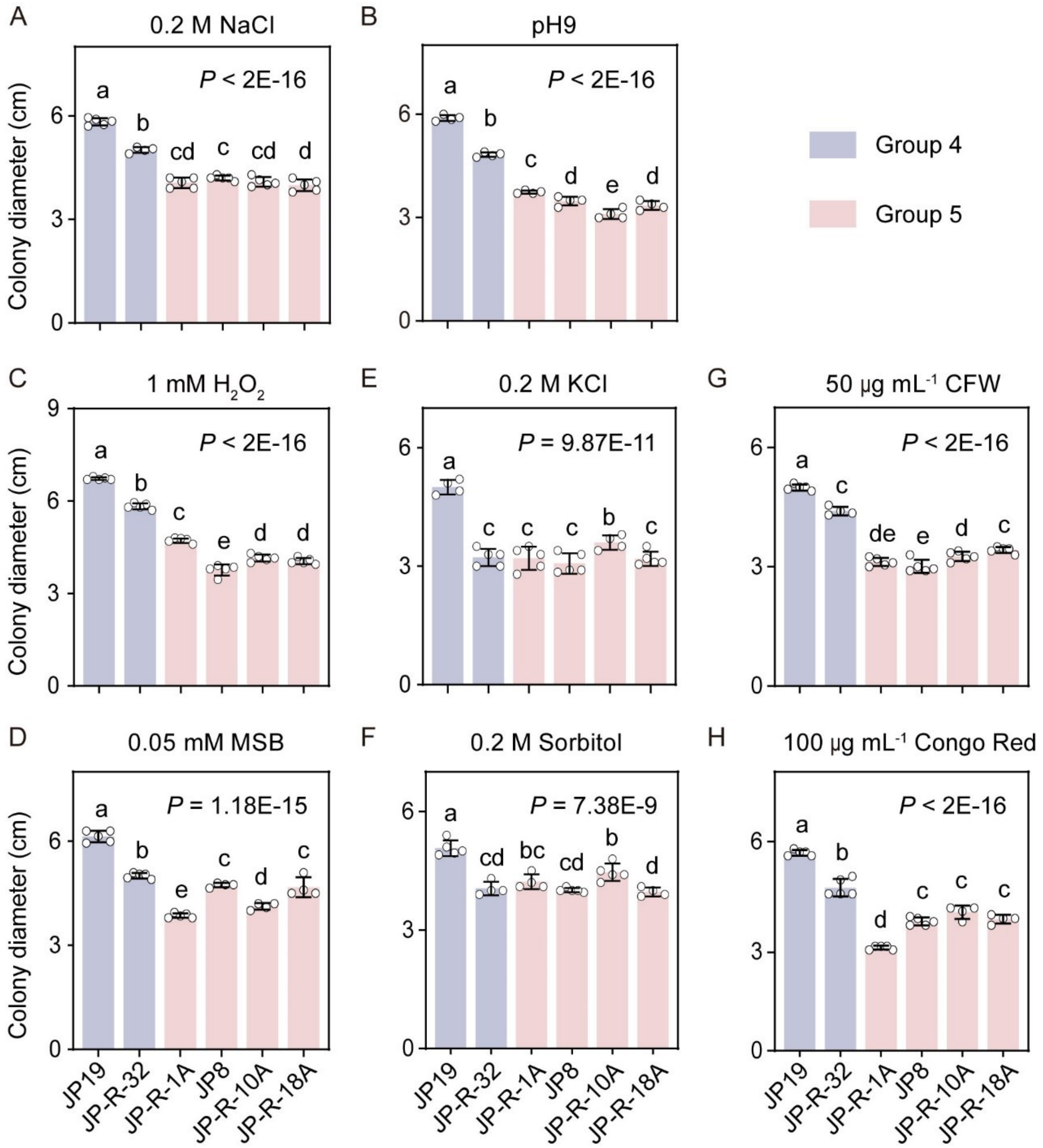

Fig. S10 Comparison of growth behavior among six diploids when exposed to a wide range of abiotic stresses. (A-B) Fungal growth under saline-alkali stress conditions (NaCl and pH). **(C-D)** The impact of oxidative stress (H_2O_2 and MSB) on growth was evaluated. **(E-F)** Growth performance was analyzed under osmotic stress conditions (KCl and sorbitol). **(G-H)** Response to cell wall stress (CFW and Congo red) was examined. Values are means ± SD for (A, C-H) (n = 5 biologically independent samples), and (B) (n = 4 biologically independent samples). Significant differences between diploids determined by the one-way analysis of variance (ANOVA) analyses. Different letters indicate statistically significant differences based on Fisher's LSD test analysis ($P < 0.05$). All data are representative of at least 4 experimental replicates. MSB, 2-methyl-1,4-naphthoquinone; CFW, calcofluor white. Source data are provided as a Source Data file.

Table S1 Proportion of TEs in seven haplotype genomes at the superfamily level.

Type		% of genome (JP19^{A})	% of genome (JP19^{B})	% of genome (JP8^{A})	% of genome (JP8^{B})	% of genome (JP11)	% of genome (JP44)	% of genome (R22)
Retro	LTR/Copia	15.10939	16.20869	14.68138	17.77061	14.2675	10.67844	12.09245
	LTR/Gypsy	12.7762	14.25498	12.50938	13.68962	16.29202	12.25131	12.52898
	LTR/Other	2.2116	2.60342	2.81181	3.03026	2.76388	0.72846	1.27344
	SINE	0.0003	0.00045	0.00033	0.00046	0.00139	0.00107	0.00026
	LINE	1.28005	2.60844	0.71291	1.96964	2.0413	1.33271	0.6843
	Other	0.0001	0	0.00021	0	0	0.00016	0.00011
	Total	31.37764	35.67598	30.71602	36.46059	35.36609	24.99215	26.57954
DNA	Academ	0.15271	0.7115	0.40686	1.19042	1.15788	0.00048	0.16557
	CACTA	1.46845	1.05463	0.68461	0.37321	0.15553	0.07877	0.51257
	Crypton	0.03341	0.13426	0.02018	0.03981	0.13589	0.01957	0.01492
	Dada	0.00184	0.00158	0.00154	0.00114	0.00127	0.00206	0.002
	Ginger	0.00256	0.00097	0.00294	0.00248	0.00176	0.00292	0.00151
	hAT	1.25494	2.25323	1.20113	1.30414	1.89605	1.35834	1.16789
	Helitron	0.01198	0.01378	0.01357	0.01619	0.01356	0.01121	0.01105
	Kolobok	0.01825	0.01334	0.01798	0.01355	0.01223	0.0178	0.15346
	Mutator-like	0.2517	0.38817	0.11387	0.07696	0.02248	0.04593	0.01772
	P_Element	0.00982	0.00525	0.00621	0.00592	0.00488	0.00659	0.00847
	PIF-Harbinger	0.28982	0.19531	0.15638	0.12543	0.09866	0.01428	0.17103
	PiggyBac	0.00741	0.04922	0.01238	0.05636	0.00156	0.00135	0.00076
	Sola	0.00321	0.00286	0.00427	0.00204	0.0022	0.00286	0.00254
	TcMar	10.93043	14.22855	11.29587	15.10516	14.21761	10.03433	9.55667
	Other	0.21066	0.75717	0.31508	0.13788	0.97902	3.04271	0.42312
	Total	14.64719	19.80982	14.25287	18.45069	18.70058	14.6392	12.20928
Other	-	3.05916	1.54915	0.69586	0.42494	0.05862	1.47735	2.03127
Unknown	-	6.29033	4.21984	6.40499	7.34791	5.39829	6.77391	6.9645
Total	-	45.60756	50.55117	42.89912	50.36405	47.96908	41.13388	42.1175

Table S2 The subgenomic signatures of JP19 and JP8.

		Genome size (bp)	Gene content	GC density	Gene density	TE density	Exon content	Exon number per gene	Average gene length
JP19	Subgenome A	62803122	13516	0.446	0.34	0.46	38783	2.87	1573.73
	Subgenome B	67204073	15093	0.463	0.35	0.51	41638	2.76	1550.09
JP8	Subgenome A	58696276	13307	0.449	0.35	0.43	38319	2.88	1574.42
	Subgenome B	68471488	15454	0.463	0.35	0.51	42557	2.75	1551.24

Note: the non-anchored scaffolds (a total of length of 1.86 Mb and 2.35 Mb in JP19 and JP8, respectively) were excluded from this analysis.

Table S3 GO term enrichment analysis for the NDE-TUR genes from JP19 under 0.3 M NaCl.

ID	Description	p.adjust	Count
GO:0046470	phosphatidylcholine metabolic process	0.001766785	9
GO:0006629	lipid metabolic process	0.002012483	53
GO:0046486	glycerolipid metabolic process	0.007065408	22
GO:0010876	lipid localization	7.07E-03	14
GO:0044255	cellular lipid metabolic process	0.007065408	43
GO:0006658	phosphatidylserine metabolic process	7.07E-03	6
GO:0015850	organic hydroxy compound transport	0.007065408	9
GO:0006656	phosphatidylcholine biosynthetic process	7.07E-03	7
GO:0006650	glycerophospholipid metabolic process	0.011606052	19
GO:0097164	ammonium ion metabolic process	0.018976484	9
GO:0016042	lipid catabolic process	0.020982809	16
GO:0006869	lipid transport	0.022210787	12
GO:0051403	stress-activated MAPK cascade	0.022210787	7
GO:0071214	cellular response to abiotic stimulus	0.022210787	18
GO:0104004	cellular response to environmental stimulus	0.022210787	18
GO:0006644	phospholipid metabolic process	0.023769737	22
GO:0015980	energy derivation by oxidation of organic compounds	0.041563027	18
GO:0015849	organic acid transport	0.041563027	16
GO:0006812	cation transport	0.04833329	25

Note: the *P* values calculated by one-sided hypergeometric test and corrected for multiple testing using the Benjamini–Hochberg approach.

Table S4 The expression of the NDE-TUR-enriched gene from JP19 related to cation transport was compared with that of its parent isolates under 0.3 M NaCl.

Gene ID	JP19 FPKM (Mean)	R22 FPKM (Mean)	JP11 FPKM (Mean)
JP11_08852	906.94	135.73	73.52
JP11_07391	273.83	49.30	41.26
JP11_07390	251.32	40.18	43.72
JP11_04261	352.99	120.77	84.12
JP11_04633	2555.86	465.49	635.46
JP11_05014	39.66	7.94	13.44
JP11_10345	163.56	71.07	64.47
JP11_13718	290.44	111.30	124.17
JP11_10869	156.62	64.14	67.25
JP11_04416	83.10	34.04	36.92
JP11_00895	559.20	165.58	254.01
JP11_05292	543.43	245.78	258.56
JP11_15229	50.28	23.85	24.01
JP11_10995	138.45	61.15	67.34
JP11_08395	46.66	29.38	23.71
JP11_06000	874.60	295.75	456.86
JP11_04433	180.42	55.21	95.62
JP11_08732	363.77	133.95	208.05
JP11_02440	66.63	43.35	38.47
JP11_10096	81.59	40.04	49.35
JP11_11135	3.78	2.39	2.34
JP11_10127	445.71	256.16	275.81
JP11_14784	149.69	65.74	95.00
JP11_06496	49.49	29.42	31.41
JP11_15051	52.42	30.91	33.38

Table S5 General mapping information for the newly sequenced 18 isolates of *Laburnicola rhizohalophila*

Sample	Total	Mapped	Mapped reads (%)	Genome coverage (%)	Sequencing depth
JP_R2018DY_1A	50143516	45271895	90.28	97.8	103.1
JP_R2018DY_2A	50977010	44806778	87.9	81.36	115.48
JP_R2018DY_3A	50711760	47045825	92.77	89.59	115.23
JP_R2018DY_5A	51046720	45324725	88.79	81.34	116.57
JP_R2018DY_6A	51052369	44837722	87.83	81.1	114.93
JP_R2018DY_7A	51176754	45877029	89.64	82.1	117.46
JP8	50147432	45634444	91	97.78	103.88
JP_R2018DY_9A	51021024	44846593	87.9	82.89	113.98
JP_R2018DY_10A	50137042	45404466	90.56	97.81	103.55
JP11	50918296	45293126	88.95	81.73	117.08
JP_R2018DY_12A	49179829	46854480	95.27	96.57	114.81
JP_R2018DY_13A	49205752	47397094	96.32	96.77	116.98
JP_R2018DY_16A	50985547	44677544	87.63	81.78	115.39
JP_R2018DY_17A	51104775	45276793	88.6	81.58	116.69
JP_R2018DY_18A	50076466	45277346	90.42	97.82	102.23
JP_R2018DY_19A	49102680	45714765	93.1	96.46	111.95
JP_R2018DY_20A	51203423	45387384	88.64	81.48	115.41
JP_R2018DY_21A	50865394	45620154	89.69	80.4	119.76

Table S6 Primer pairs targeting three representative contigs with dense heterozygous SNPs used to validate the origin of the diploids.

SEQ1	Forward: 5'-GCGTTTGCCTCCTTC-3'
	Reverse: 5'-TTCCTCTGGCTGTTCC-3'
SEQ2	Forward: 5'-CGCTTCCGCTCATACT-3'
	Reverse: 5'-ACGCAACATAGCCTCC-3'
SEQ3	Forward: 5'-TAAATATTGCGAGATCCGC-3'
	Reverse: 5'-GGGCTCAGAGTCATTTGCT-3'
