# Supplementary material for: Intraspecific diploidization of a halophyte root fungus drives heterosis
Source: Nat Commun. 2024 Jul 12;15:5872. doi: 10.1038/s41467-024-49468-7 (PMC11245560; doi:10.1038/s41467-024-49468-7)
Supplement: Supplementary file 3 — Description of Additional Supplementary Files [file 41467_2024_49468_MOESM3_ESM.pdf]

Description of Additional Supplementary Files

File Name: Supplementary Data 1
Description: The expression of the NDE-TUR-enriched gene from JP19 related to membrane phospholipid biosynthesis was compared with that of its parents under 0.3 M NaCl

File Name: Supplementary Data 2
Description: Summary of 47 isolates of *L. rhizohalophila* used in this work
