# Supplementary material for: Intraspecific diploidization of a halophyte root fungus drives heterosis
Source: Nat Commun. 2024 Jul 12;15:5872. doi: 10.1038/s41467-024-49468-7 (PMC11245560; doi:10.1038/s41467-024-49468-7)
Supplement: Supplementary file 6 — Reporting Summary [file 41467_2024_49468_MOESM6_ESM.pdf]

nature portfolio

Corresponding author(s): Zhilin Yuan, Peng Zhou, Francis M. Martin

Last updated by author(s): Apr 12, 2024

Reporting Summary

Nature Portfolio wishes to improve the reproducibility of the work that we publish. This form provides structure for consistency and transparency in reporting. For further information on Nature Portfolio policies, see our Editorial Policies and the Editorial Policy Checklist.

Statistics

For all statistical analyses, confirm that the following items are present in the figure legend, table legend, main text, or Methods section.

n/a	Confirmed	
☐	☒	The exact sample size (*n*) for each experimental group/condition, given as a discrete number and unit of measurement
☐	☒	A statement on whether measurements were taken from distinct samples or whether the same sample was measured repeatedly
☐	☒	The statistical test(s) used AND whether they are one- or two-sided<br>*Only common tests should be described solely by name; describe more complex techniques in the Methods section.*
☒	☐	A description of all covariates tested
☐	☒	A description of any assumptions or corrections, such as tests of normality and adjustment for multiple comparisons
☐	☒	A full description of the statistical parameters including central tendency (e.g. means) or other basic estimates (e.g. regression coefficient) AND variation (e.g. standard deviation) or associated estimates of uncertainty (e.g. confidence intervals)
☐	☒	For null hypothesis testing, the test statistic (e.g. *F*, *t*, *r*) with confidence intervals, effect sizes, degrees of freedom and *P* value noted<br>*Give P values as exact values whenever suitable.*
☒	☐	For Bayesian analysis, information on the choice of priors and Markov chain Monte Carlo settings
☒	☐	For hierarchical and complex designs, identification of the appropriate level for tests and full reporting of outcomes
☒	☐	Estimates of effect sizes (e.g. Cohen's *d*, Pearson's *r*), indicating how they were calculated

Our web collection on statistics for biologists contains articles on many of the points above.

Software and code

Policy information about availability of computer code

Data collection	This study did not use any commercial, open source and custom code to collect data.
Data analysis	Genomics processing: BWA v0.7.17; SAMtools v1.3; BCFtools v1.10.2; TreeBest v1.9.2;SplitsTree v4.14.4; hifiasm v0.15.4-r342; 3D-DNA; Juicerbox; MAKER v2.31.9; BUSCO v5.beta.1; OrthoDB fungi v10; TRF v4.09; RepeatModeler v2.0.2a; RepeatScout v1.0.6;Piler v1.0; LTR_FINDER v1.07; RepeatMasker v4.1.2; Repbase v21.01; Dfam v3.0; FCS Express v7.0; Jellyfish v2.1.4; Smudgeplot v0.2.5; GenomeScope v2.0; MUMmer v4.0.0; JCVI v0.5.7; OrthoFinder v2.3.11; MUSCLE v3.8.31; RAxML v8.2.4; FACET toolkit; BioXM v2.6; Statistical analyses: R statistical programming language version 3.6.3.Packages: ggplot2; stats; agricolae. The source code implementing the analyses in this manuscript is available on Github (https://github.com/TaoFu123/FungiAnalysis.git) and Gitee (https://gitee.com/orionzhou/rnaseq/).

For manuscripts utilizing custom algorithms or software that are central to the research but not yet described in published literature, software must be made available to editors and reviewers. We strongly encourage code deposition in a community repository (e.g. GitHub). See the Nature Portfolio guidelines for submitting code & software for further information.

Data

Policy information about availability of data

All manuscripts must include a data availability statement. This statement should provide the following information, where applicable:

- Accession codes, unique identifiers, or web links for publicly available datasets
- A description of any restrictions on data availability
- For clinical datasets or third party data, please ensure that the statement adheres to our policy

The Illumina sequencing data for the 18 isolates have been deposited in the Sequence Read Archive (SRA) at NCBI under the accession numbers SRR23813528-SRR23813545. PacBio HIFI reads for JP19, JP8, JP44, and JP11 have been deposited in SRA under accession numbers SRR23908958-SRR23908961. The Hi-C reads from the five representative isolates have been deposited in SRA under the accession numbers SRR23901851-SRR23901855. Furthermore, the RNA-seq reads from the 60 libraries have been deposited in SRA under accession numbers SRR23343132-SRR23343191. The assembled genomes have been deposited in whole genome shotgun (WGS) under accession numbers JAULRH000000000, JAULRI000000000, JAULRJ000000000, JAUOZT000000000, and JAUPCS000000000. The sequencing data of additional 114 isolates have been deposited in the SRA under accession numbers SRR26626062-SRR26626175. All data have been submitted under the BioProject accession numbers PRJNA517533 (https://www.ncbi.nlm.nih.gov/bioproject/PRJNA517533) and PRJNA931727 (https://www.ncbi.nlm.nih.gov/bioproject/PRJNA931727). Annotations of genomes from the five isolates are provided in the Supplementary Materials. Source data are provided with this paper.

Research involving human participants, their data, or biological material

Policy information about studies with human participants or human data. See also policy information about sex, gender (identity/presentation), and sexual orientation and race, ethnicity and racism.

Reporting on sex and gender	not applicable
Reporting on race, ethnicity, or other socially relevant groupings	not applicable
Population characteristics	not applicable
Recruitment	not applicable
Ethics oversight	not applicable

Note that full information on the approval of the study protocol must also be provided in the manuscript.

Field-specific reporting

Please select the one below that is the best fit for your research. If you are not sure, read the appropriate sections before making your selection.

☐ Life sciences ☐ Behavioural & social sciences ☒ Ecological, evolutionary & environmental sciences

For a reference copy of the document with all sections, see nature.com/documents/nr-reporting-summary-flat.pdf

Ecological, evolutionary & environmental sciences study design

All studies must disclose on these points even when the disclosure is negative.

Study description	In the present study, we found that a naturally occurring recombinant hybrid genotype exhibited enhanced growth under various abiotic stresses. This observation motivated us to investigate how the ploidy change in the DSE fungus improves its fitness by comparing the gene expression patterns in each hybrid-parent triad under different growth conditions. In order to solve the above scientific problems and ensure that our samples are representative of the local population, we continuously expand the L.rhizohalophila population that collected from common halophyte Suaeda salsa roots along the coastal area under different salt-alkali condition in Dongying, Shandong province, China. 29 individuals were collected in July 2013 and July 2014, and June 2015, as previously reported by Yuan et al. (2021), while the remaining 18 individuals were collected in July 2018. To ensure that the most basic statistical principles were met, we randomly chose 8-10 plots per year and replicated at least 20 plants in each plot. A total of 47 L.rhizohalophila isolates collected and the NJ tree revealed the presence of five distinct genetic clades, designated as Groups 1-5. JP19 and JP8 from group 4 and 5 could be considered as intraspecific diploid hybrids. And we used all samples (n=47) to investigate how the ploidy change in the DSE fungus improves its fitness by comparing the gene expression patterns in each hybrid-parent triad under different growth conditions.
Research sample	This study includes 47 L. rhizohalophila isolates from the roots of Suaeda salsa which is native to saline soils in the Yellow River Delta (Dongying City, Shandong Province, China). Among the 47 isolates of L. rhizohalophila, 29 individuals were collected in July 2013 and July 2014, and June 2015, as previously reported by Yuan et al. (2021). The remaining 18 individuals were collected in July 2018.
Sampling strategy	In order to ensure that our samples are representative of the local population. We constantly expand the L.rhizohalophila population for four year from the Suaeda salsa roots along the coastal area under different salt-alkali condition randomly in Dongying (N37°23′

	43′′, E118 °55′25′′), Shandong province, China. 29 individuals were collected in July 2013 and July 2014, and June 2015, as previously reported by Yuan et al. (2021). The remaining 18 individuals were collected in July 2018. To ensure that the most basic statistical principles were met, we randomly chose 8-10 plots per year and replicated at least 20 plants in each plot. At each time, the sampling sites occupy approximately 30 square kilometers, which can represent the population of L.rhizohalophila in this area. There is no sample size calculation were performed. We obtained a total of 47 L.rhizohalophila isolates and we used all (n=47) samples collected from Suaeda salsa to represent full diversity of L.rhizohalophila population.
Data collection	The data collection period runs from July 2013 to July 2018. All the sampling sites collectively cover an area of approximately 30 square kilometers, adequately representing the population of L. rhizohalophila in this region. Genomic data and of 29 individuals were publicly accessible, as previously reported by Yuan et al. (2021). Then, we collected living cultures and genomic data from an additional 18 isolates of L. rhizohalophila. Fungal isolation was conducted from sterile root tissues onto 1% malt extract agar (MEA, Oxoid) by Zhongfeng Li and Zhiyong Zhu.Genome resequencing was performed using MGISEQ libraries with an insert size of 350 bp. The MGISEQ-2000 instrument was used for paired-end sequencing with a read length of 150 bp (BGI, Wuhan, China). To obtain high-quality genome sequences for five representative isolates from each population (JP19, JP8, R22, JP44, and JP11), high-throughput genome sequencing was conducted using PacBio Sequel II and the circular consensus sequencing (CCS) mode at Novogene (Tianjin, China). It should be noted that the chromosomal-level assembly of R22 has already been published by He and Yuan in 2021. We analyzed the expression profiles of these two diploids and three haploids using RNA-seq data under four fungal growth conditions, including three gradients of salt stress (0 M NaCl, 0.3 M NaCl and 0.8 M NaCl) in vitro and a symbiotic status in planta. Three independent biological replicates were used for each growth condition, resulting in 60 libraries.
Timing and spatial scale	The sample collection period extends from July 2013 to July 2018. Among collected 47 L. rhizohalophila isolates, 29 individuals were collected in July 2013 and 2014, and in June 2015. And in July 2018 we collected the remaining 18 individuals. At each time, the sampling sites cover approximately 30 square kilometers, effectively representing the population of L. rhizohalophila in this area.
Data exclusions	No data excluded, Supplemental Data Files contain all datasets generated
Reproducibility	We confirm that all attempts at replications were successful.
Randomization	We selected roots of Suaeda salsa randomly in this study.
Blinding	Data collection and data analysis were not blindly, because samples in this study were collected from field coastal area, and non-blind design would not affect the experimental results.

Did the study involve field work? ☒ Yes ☐ No

Field work, collection and transport

Field conditions	Suaeda salsa roots were collected along the coastal area. The area is low and flat, with mean annual temperature of 12.9°C, and with mean annual precipitation of 596.9 mm, it is a continental monsoon climate in the north temperate zone.
Location	Yellow River Delta in Dongying City, Shandong Province, China (N37°23′43′′, E118 °55′25′′), with an average altitude of 4.5m.
Access & import/export	The first sampling in July 2013, we accessed this field by connecting the manager Dr.Rongsong Zou of the Yellow River Delta Comprehensive Test Center, Chinese Academy of Forestry. The samples collected in July 2013 and June 2014, June 2015 and July 2018 used in our study were all permitted.<br>We imported root samples into our laboratory from field by using cool box. It is safe and does not affect society activity.
Disturbance	There is no disturbance during our sampling.

Reporting for specific materials, systems and methods

We require information from authors about some types of materials, experimental systems and methods used in many studies. Here, indicate whether each material, system or method listed is relevant to your study. If you are not sure if a list item applies to your research, read the appropriate section before selecting a response.

Materials & experimental systems

n/a	Involved in the study
☒	☐ Antibodies
☒	☐ Eukaryotic cell lines
☒	☐ Palaeontology and archaeology
☒	☐ Animals and other organisms
☒	☐ Clinical data
☒	☐ Dual use research of concern
☐	☒ Plants

Methods

n/a	Involved in the study
☒	☐ ChIP-seq
☐	☒ Flow cytometry
☒	☐ MRI-based neuroimaging

Plants

Seed stocks

We did not collect the the plant seeds in our work.

Novel plant genotypes

There are no novel plant genotypes of Suaeda salsa and Populus tomentosa.

Authentication

n/a

Flow Cytometry

Plots

Confirm that:

☒ The axis labels state the marker and fluorochrome used (e.g. CD4-FITC).

☒ The axis scales are clearly visible. Include numbers along axes only for bottom left plot of group (a 'group' is an analysis of identical markers).

☒ All plots are contour plots with outliers or pseudocolor plots.

☒ A numerical value for number of cells or percentage (with statistics) is provided.

Methodology

Sample preparation

Prior to flow cytometry analysis, we prepared protoplasts from the five isolates. The fungus was inoculated into 100 mL of fresh PDB liquid medium in a 250 mL flask and shaken at 180 rpm at 20°C for 2 d. Mycelia were collected, washed twice with 0.6 M KCl (pH5.8) and lysed with a combination of 1% lysing enzyme (Sigma-Aldrich, USA), 2% cellulase (Sangon Biotech, China), and 2% snailase (Sangon Biotech, China). A protoplast suspension (3-5×106 mL-1) was prepared. Flow cytometry analysis was conducted as follows: 1 mL protoplast suspension was subjected to centrifugation for 10 min at 14,000 × g. After removing the supernatant, protoplasts were fixed in a mixture of methanol: acetic acid (3:1, v/v), 10% dimethyl sulfoxide (v/w), and 0.1% Triton X/100 (v/w) for 1 h at 4°C. Subsequently, the samples were chopped using a razor blade in Tris/MgCl2 buffer (pH7.5) with 0.1 mg mL-1 RNase A (Sangon Biotech, China). The suspension containing released nuclei was filtered through a 20 µm nylon filter to eliminate large debris and then incubated at 37°C for 15 min. Nuclei were stained with 50 µg mL-1 propidium iodide (PI) (Fluka, Glossop, England) and immediately analyzed using an LSRII FACS machine (Becton Dickinson, NJ, USA).

Instrument

LSRII FACS machine (Becton Dickinson, NJ, USA)

Software

FCS Express v7.0 (De Novo Software, Los Angeles, CA, USA)

Cell population abundance

Each measurement consisted of 10,000 nuclei, and each isolate was measured three times.

Gating strategy

We set the electronic trigger to the FL2 channel (the 580/42 optical filter in front of the 2nd PMT collecting yellow orange emission from the initial 488nm laser line excitation) to focus solely on events that are propidium iodide (PI) positive, i.e. those that contain nucleic acid. We used an appropriate internal sizing control (Arabidopsis thaliana and Fusarium pseudograminearum) of known genome size to set the detector voltage on the 580/42 detector to approximately 500V, which enabled identifying small (L. rhizohalophila haploid isolates) and large (L. rhizohalophila diploid isolates) genome sizes simultaneously on scale.

☐ Tick this box to confirm that a figure exemplifying the gating strategy is provided in the Supplementary Information.
